# Sclerosing Encapsulating Peritonitis: A Rare Cause of Intestinal Obstruction

**DOI:** 10.7759/cureus.15291

**Published:** 2021-05-28

**Authors:** Mohamed H Yusuf

**Affiliations:** 1 General Surgery, Salmaniya Medical Complex, Manama, BHR

**Keywords:** sclerosing encapsulating peritonitis, bowel obstruction, abdominal cocoon, abdominal pain, case report

## Abstract

Sclerosing encapsulating peritonitis (SEP) is a rare clinical entity that may cause small bowel obstruction. It is characterized by a thick fibrocollagenous cocoon-like membrane. Surgical intervention is the mainstay of treatment. A 36-year-old Pakistani man presented with recurrent attacks of colicky abdominal pain, distention, vomiting, and constipation. Abdominal CT revealed a thick enhanced membrane forming a sac that contained clusters of small intestinal loops. Exploratory laparotomy showed a thick membrane containing the small bowel and extensive inter-loop adhesions. The sac underwent decortication and excision, inter-loop adhesions were released, and an appendectomy was performed. The patient tolerated the procedure and was discharged in good condition.

## Introduction

Sclerosing encapsulating peritonitis (SEP) is a rare clinical entity of unclear etiology that is characterized by a thick fibrocollagenous membrane that partially or completely encases the small intestine and/or the colon. It may lead to acute, subacute, or chronic episodes of intestinal obstruction. It can be idiopathic or secondary to a variety of underlying conditions [1]. Herein, we report a patient with recurrent episodes of abdominal pain and small bowel obstruction due to SEP.

## Case presentation

A 36-year-old Pakistani male presented with colicky abdominal pain, distention, bilious vomiting, constipation for three days, and a history of similar attacks over the last three months resolved spontaneously.

He had no history of constitutional symptoms and no significant past surgical or medical history. His vital signs were within normal limits. Abdominal examination showed central abdominal distension, tender central abdominal mass, guarding, and hyperactive bowel sounds. However, the abdomen was soft and lax with no clinical signs of peritonitis. Digital rectal examination was unremarkable.

The white blood cell count was 18,000/µL with a neutrophil predominance (83.1%) and the erythrocyte sedimentation rate was 25 mm/h (0-20). Hemoglobin level, platelet count, quantitative C-reactive protein, serum electrolytes, urea, creatinine level, and liver function tests were within normal limits.

**Figure 1:**
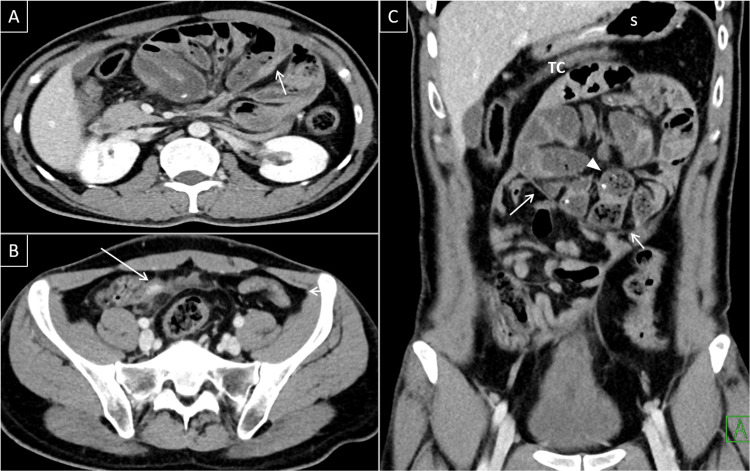
Abdominal CT images. Axial (A and B) and coronal (C) contrast-enhanced CT images showing a cluster of dilated small bowel loops that are drawn into the center of the abdominal cavity, pushing the stomach (S) and transverse colon (TC) superiorly. Note enhanced thickened membrane surrounding the bowel loops (arrows) and the fecalization of small bowel content (arrowhead in C). Thickened wall appendix with surrounding fat stranding (long arrow in B).

Frontal chest radiograph demonstrated peripherally located opacities suggestive of COVID-19 infection, which was confirmed by viral PCR. An abdominal CT scan revealed a thick enhanced membrane forming a sac that contained a cluster of small intestinal loops giving the appearance of a cocoon, fecalization of the small bowel content, displacement of both stomach and transverse colon superiorly. There was no evidence of intra-abdominal collection (Figure 1).

**Figure 2:**
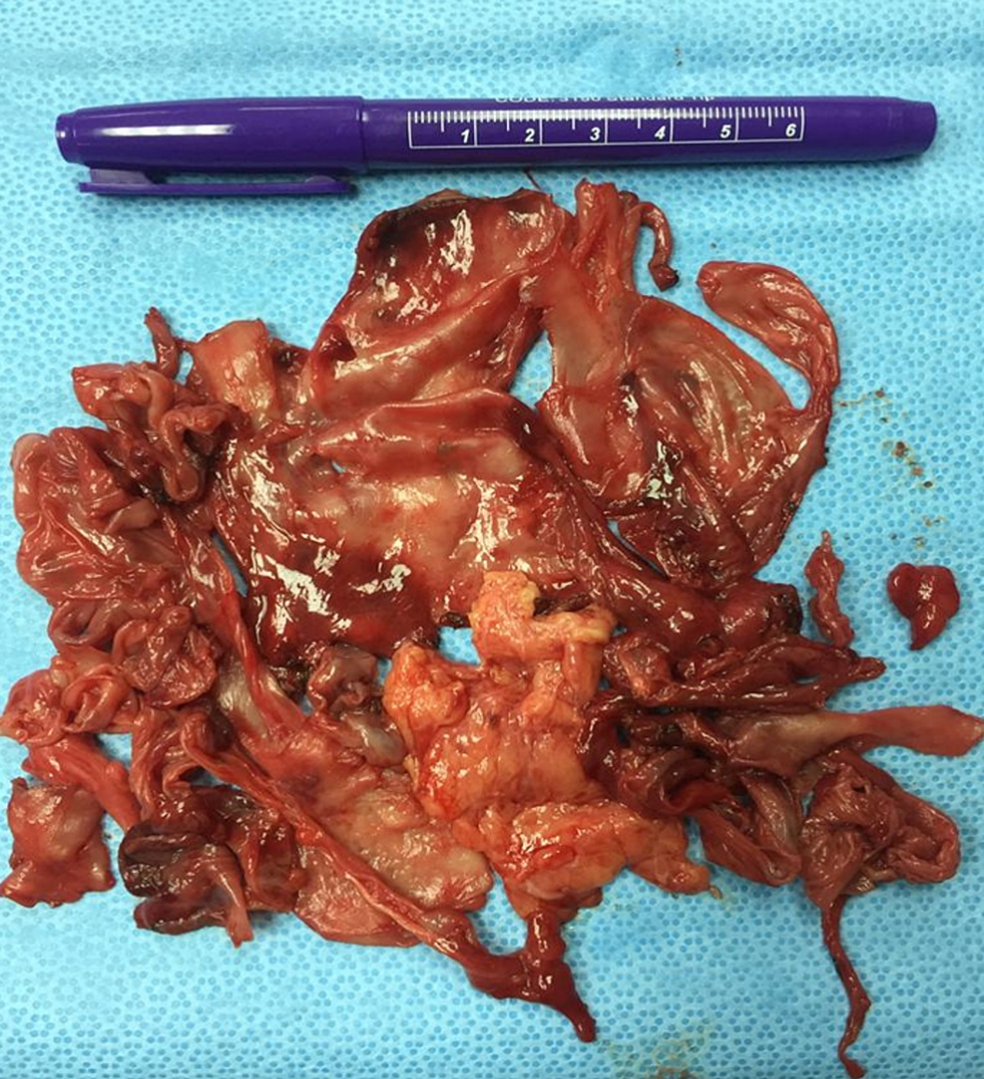
Gross pathology image. Gross pathology image showing the resected membrane around the small intestine.

Exploratory laparotomy through midline incision showed thick membrane containing the small bowel, extensive inter-loop adhesion, and appendicular fecolith. No evidence of abscess or fluid collection was noted. The sac was decorticated and excised followed by adhesiolysis and appendectomy (Figure 2).

Stomach and bowel exploration did not show any perforation. Peritoneal fluid cultures were positive for Streptococcus constellatus and Staphylococcus epidermidis. Mycobacterium culture (acid-fast culture) did not show growth after eight weeks. Histopathology revealed dense fibrocollagenous tissue associated with a non-specific chronic inflammatory reaction, no malignant cells were identified, and the appendix showed lymphoid follicular hyperplasia. No granuloma was observed on histopathological examination. The patient tolerated the procedure and discharged in good condition seven days after the operation. The patient was seen in the clinic with no active complaint or similar attack.

## Discussion

SEP was first reported by Owtschinnikow in 1907 who used the term “peritonitis chronica fibrosa encapsulate” [1]. In 1978, Foo et al. coined the term “Abdominal Cocoon Syndrome” to describe the fibrous capsule seen during laparotomy in 10 young adolescent girls with this condition [2].

The classic description of this condition in adolescent females from tropical and subtropical regions led to the hypothesis that the etiology of this condition could be a result of retrograde menstruation [2]. However, this theory has fallen out of favor as the condition has been described in men and premenopausal women as well. Interestingly, a review of the literature on SEP consisting of 118 cases has shown that this condition is twice as common in males as in females [3].

There is an inflammatory reaction in the peritoneal membrane that may be related to the increased release of cytokines causing copious fibrin deposition on the peritoneum covering the abdominal viscera [4]. Some research suggested that this condition arises due to subclinical primary viral peritonitis and immunological reactions [5]. Despite various hypotheses, the exact explanation of this condition is still not well understood.

SEP is more often associated with other conditions and factors than to be of idiopathic etiology. The most well-known association is noted in patients with renal impairment on continuous ambulatory peritoneal dialysis, with a reported incidence of 13.6 per 1000 patient-years [6]. It has been reported in patients with a history of peritonitis, peritoneal shunting, and intraperitoneal chemotherapy. Other associations include liver cirrhosis, post-liver transplantation, sarcoidosis, systemic lupus erythematosus, endometrial cysts, and ovarian tumors [3]. Certain medications have been suggested as causative factors, including methotrexate and prolonged use of beta-blocker therapy with the withdrawn drug, practolol, being the first agent to be linked to the condition, and several other agents have been reported later [7]. In tropical regions, endemic diseases such as tuberculosis have to be excluded, as tuberculous peritonitis may mimic SEP [8].

In our case, the peritoneal fluid cultures were positive for Streptococcus constellatus and Staphylococcus epidermidis, and negative for tuberculosis. Histopathology revealed dense fibrocollagenous tissue associated with non-specific chronic inflammatory reactions, and no malignant cells were identified.

It is worth mentioning that SEP may be confused with congenital peritoneal encapsulation, which is a very rare condition. In congenital conditions, there is an accessory peritoneal lining covering the small intestine that is caused by an abnormal intestinal return to the abdominal cavity during the early stages of development [9].

It can present with recurrent ascites or a palpable abdominal mass. In some patients, it may be entirely asymptomatic and discovered during surgery for a different indication. It has three types based on the severity of involvement with type 1 involving part of the small intestine, type 2 involving the small intestine completely, and type 3 involving the colon and small intestine [3].

Patients with SEP usually present with recurrent episodes of acute, subacute, or chronic intestinal obstruction with non-specific colicky abdominal pain, nausea, vomiting, anorexia, and weight loss.

Prior to the widespread use of imaging, SEP was seen as a ‘surprise’ to the surgeon during laparotomy. However, with the current high-resolution radiological imaging, preoperative diagnosis is possible [3]. Plain radiographs of the abdomen may reveal dilated small bowel loops due to obstruction, but this does not distinguish SEP from the other more common causes. Contrast studies may demonstrate the ‘cauliflower’ sign in which there are dilated small bowel loops fixed in a cluster [3]. CT remains the best imaging modality of choice, which shows a cluster of small bowel loops encased by a soft-tissue density mantle. It may also reveal peritoneal thickening and loculated fluid collections [3, 4].

Surgery is the cornerstone of the management of SEP. Bowel resection is indicated for non-viable bowel and is associated with increased morbidity and mortality [3, 10]. In general, the prognosis of SEP after surgery is satisfactory, and excision of the thick membrane and adhesiolysis of the small intestine leads to complete recovery [3]. Moreover, the long-term postoperative prognosis of idiopathic forms is excellent, with no recurrence reported [11].

## Conclusions

SEP is a rare cause of intestinal obstruction, and the diagnosis requires a high index of suspicion. The pathogenesis of COVID-19, in this case, remains unclear and the co-existence of both conditions could be merely incidental. Surgical management of decortication and adhesiolysis is the gold standard treatment.
